# T lymphocytes facilitate brain metastasis of breast cancer by inducing Guanylate-Binding Protein 1 expression

**DOI:** 10.1007/s00401-018-1806-2

**Published:** 2018-01-19

**Authors:** Dana A. M. Mustafa, Rute M. S. M. Pedrosa, Marcel Smid, Marcel van der Weiden, Vanja de Weerd, Alex L. Nigg, Cor Berrevoets, Lona Zeneyedpour, Neibla Priego, Manuel Valiente, Theo M. Luider, Reno Debets, John W. M. Martens, John A. Foekens, Anieta M. Sieuwerts, Johan M. Kros

**Affiliations:** 1 Department of Pathology, Erasmus Medical Center, Wytemaweg 80, 3000 DR Rotterdam, The Netherlands; 2 Brain Tumor Center, Erasmus Medical Center, Rotterdam, The Netherlands; 3 Department of Medical Oncology, Erasmus MC Cancer Institute, Rotterdam, The Netherlands; 4 Department of Pathology, Optical Image Center, Erasmus Medical Center, Rotterdam, The Netherlands; 5 Department of Medical Oncology, Laboratory of Tumor Immunology, Erasmus Medical Center, Rotterdam, The Netherlands; 6 Department of Neurology, Laboratory for Neuro-Oncology, Erasmus Medical Center, Rotterdam, The Netherlands; 7 Brain Metastasis Group, Spanish National Cancer Research Center (CNIO), 28029 Madrid, Spain

**Keywords:** Brain metastasis, Blood–brain barrier, T cell response, *GBP1*

## Abstract

The discovery of genes and molecular pathways involved in the formation of brain metastasis would direct the development of therapeutic strategies to prevent this deadly complication of cancer. By comparing gene expression profiles of Estrogen Receptor negative (ER-) primary breast tumors between patients who developed metastasis to brain and to organs other than brain, we found that T lymphocytes promote the formation of brain metastases. To functionally test the ability of T cells to promote brain metastasis, we used an in vitro blood–brain barrier (BBB) model. By co-culturing T lymphocytes with breast cancer cells, we confirmed that T cells increase the ability of breast cancer cells to cross the BBB. Proteomics analysis of the tumor cells revealed Guanylate-Binding Protein 1 (GBP1) as a key T lymphocyte-induced protein that enables breast cancer cells to cross the BBB. The *GBP1* gene appeared to be up-regulated in breast cancer of patients who developed brain metastasis. Silencing of *GBP1* reduced the ability of breast cancer cells to cross the in vitro BBB model. In addition, the findings were confirmed in vivo in an immunocompetent syngeneic mouse model. Co-culturing of ErbB2 tumor cells with activated T cells induced a significant increase in *Gbp1* expression by the cancer cells. Intracardial inoculation of the co-cultured tumor cells resulted in preferential seeding to brain. Moreover, intracerebral outgrowth of the tumor cells was demonstrated. The findings point to a role of T cells in the formation of brain metastases in ER- breast cancers, and provide potential targets for intervention to prevent the development of cerebral metastases.

**Electronic supplementary material:**

The online version of this article (10.1007/s00401-018-1806-2) contains supplementary material, which is available to authorized users.

## Introduction

Breast cancer is among the tumors that most frequently metastasize to brain [30, 37, 38, 40, 43, 49, 57]. The appearance of metastases in brain invariably defines the terminal stage of disease for women suffering from disseminated breast cancer and the prevention of cerebral spread would significantly improve their survival [44]. The formation of cerebral metastases depends on the capability of circulating tumor cells to successfully penetrate the blood–brain barrier (BBB). Various genes and pathways have been associated with seeding of breast cancer cells to various organs and the rise of brain metastases in particular [6, 32, 47, 52, 54]. Several associations between innate features of the primary tumors and their propensity to intracerebral seeding have been reported. HER2-enriched (HER2 +) and triple-negative (TNBC) primary tumors are at higher risk for developing brain metastases relative to the hormone receptor-positive tumors [17, 18, 29, 53, 59]. So far, *ST6GALNAC5* is the only specific gene that was found to mediate the formation of brain metastases of a human breast cancer-derived cell line when injected in mice. Moreover, its expression in human breast cancer samples appeared to be associated with the occurrence of cerebral metastases [3]. However, the identification of pathways associated with brain metastasis is necessary to elucidate the mechanisms of crossing the BBB and developing strategies to prevent the formation of brain metastasis.

Here, we sought pathways specifically involved in the formation of cerebral metastases of breast cancer by comparing RNA expression profiles of primary ER- breast cancer samples of patients who developed cerebral metastases, with those who developed metastasis to other organs but not to brain. We discovered that the T cell response is crucial for the development of brain metastases. In both in vitro studies using a BBB model and in vivo studies using a mouse model, T cells appear to change the expressional profiles of the breast cancer cells and facilitate their passage through the BBB. Guanylate-binding protein 1 (GBP1) is prominent among the involved proteins and its expression appears to be upregulated in the primary tumor specimens. Silencing of *GBP1* significantly decreased the ability of breast cancer cells to cross the BBB. The involvement and specific action of T lymphocytes in the process of cerebral metastasis is novel, and opens new therapeutic opportunities for preventing tumor cells to enter the brain.

## Methods

### Tissue sample selection

To identify genes and pathways involved in the formation of brain metastasis, we exclusively used specimens of primary tumors, and did not use specimens of metastatic sites. Fresh frozen (FF) tissue specimens of 22 primary breast cancer patients who developed metastasis to brain and/or to other organs were selected. Two groups of samples were compared; those from patients who had developed brain metastasis (exclusively or in addition to a maximum of 2 organs; *n* = 13) and those from women who developed metastases to a maximum of three organs (*n* = 9). None of the 22 patients had received adjuvant therapy (chemo- or hormonal therapy) prior to developing metastases, all samples were ER- and none of the patients had more than three metastatic sites. The relevant clinical data are provided in Table 1. In addition, we used 20 primary breast cancer samples for independent validations from patients of whom 13 developed brain metastasis. This study was approved by the Medical Ethics Committee of the Erasmus Medical Center, Rotterdam, The Netherlands (MEC 02·953) and performed in adherence to the Code of Conduct of the Federation of Medical Scientific Societies in The Netherlands (http://www.fmwv.nl/).

**Table 1:**
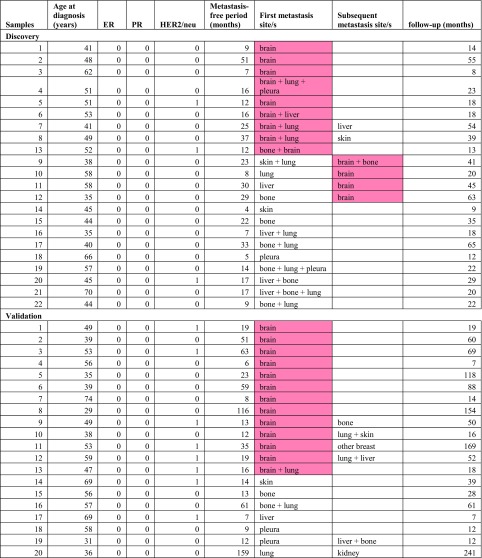
Clinical information

Age at diagnosis in years; metastasis-free periods and follow-up in months. For ER, PR, HER2/neu status: 0 = negative, 1 = positive

### Morphological assessment

5 µm H&E sections from each sample were prepared before and after sectioning for RNA isolation. To ascertain the origin of the tumor, the percentages of the invasive tumor cells, inflammatory infiltrates and the presence of necrosis were taken into consideration (JMK).

### RNA extraction and purification and quality control

Total RNA from FF tissue samples was extracted from 20 to 30 sections of 30 μm (depending on the size of the sample) using RNABee reagent according the supplier’s instructions (Campro Scientific, Veenendaal, The Netherlands) [50]. Following isolation, RNA was stored in RNase/DNase-free water at − 80 °C. The quantity and quality of the isolated RNA was assessed by nanodrop. Samples were excluded if the yield did not reach the minimum requirement of 1000 ng.

### Gene expression profiling

Illumina Whole-Genome cDNA-mediated Annealing, Selection, extension and Ligation (WG-DASL) assay was used to profile the samples. In the assay, 24,526 annotated transcripts corresponding to 18,391 unique genes are measured. The WG-DASL assay was performed according to the manufacturer’s instructions with an input of 500 ng total RNA. To monitor the assay performance and to evaluate the inter-assay BeadChip variability between the experiments, an inner-assay control consisting of 500 ng total RNA pooled from RNA isolated from several cultured breast cancer cell lines was used in each experiment [36].

### Data analysis

Scanned data were uploaded into GenomeStudio software version 2011.1 via the WG-DASL gene expression module for further analysis. The average signal, detection *P* value, bead standard error and average beads were used to quantile normalize the data in the statistical language R (www.r-project.org) using the “Lumi” package [11].

To identify significantly differentially expressed genes, three steps were followed: sample exclusion criterion, reliable probe selection and gene expression comparisons. Sample exclusion criterion and probe selection method were described previously [36]. For the gene expression comparison, Biometric Research Branch ArrayTools (BRB-array tool (V4.3.1)) was used [51]. Within BRB, the 4150 most reliable probes for FF samples were exposed to the class comparison algorithm to identify differentially expressed genes with a maximum *P* value of 0.05 after 10,000 permutations multiple correction to determine significance.

### Pathway analysis

Pathway analysis was done by two different methods. Firstly, the differentially expressed genes (resulted from the gene expression comparisons of samples with brain metastasis compared to samples with metastasis to other organs) was submitted to Ingenuity Pathway Analysis (Ingenuity, Mountain View, CA). Secondly, all reliably profiled genes were submitted to the Global Test [16] (version 4.4.0) to associate Biocarta pathways (www.biocarta.com) to the groups of samples metastasizing to brain or to other organs. The R version 2.4.1 (www.r-project.org) was used to run the Global Test package. A *P* value for a pathway was considered significant when the univariate *P* value of the test as well as the *P* value calculated by 1000 re-samplings were both < 0.05. In addition, The lists of differentially expressed probes were uploaded into the function annotation tool DAVID version 6.7 [9] to functionally annotate the differentially expressed genes. DAVID was used with the data basses and settings that are preselected by default. Statistical analyses and multiple testing correction procedures are those included in the DAVID analyses [21].

### In vitro BBB model

#### Tissue culture procedures

Human umbilical vein endothelial cells (HUVECs, ScienCell) were cultured in endothelial cell medium (ECM, ScienCell) supplemented with endothelial cells growth factors, 5% fetal bovine serum and Penicillin/Streptomycin. HUVECs were used between passage 2 and 5. Human astrocytes (ScienCell) were cultured in astrocytes medium (AM, ScienCell) supplemented with astrocyte growth factors, 2% fetal bovine serum and Penicillin/Streptomycin. Human astrocytes were used between passage 2 and 5. No further authentication was performed for this study for HUVECs and human astrocytes. Three breast cancer cell lines which had proven to be able to cross the BBB were used: MDA-MB-231, MDA-MB-231-BM (a breast cancer cell line that metastasize specifically to brain) [3] and SUM159PT [45]. The characterization of the breast cancer cell lines are summarized (Supplementary Table 1). The tumor cell lines and normal fibroblasts (isolated from non-cancerous tissue) were previously characterized [19, 46], and were cultured in RPMI medium supplemented with l-glutamine, 10% fetal bovine serum and Penicillin/Streptomycin. T cells were isolated, activated and transduced as described previously [24, 55]. Two types of T cells were used: T cells that were transduced with an empty vector, referred to as “T cells”, and T cells that were transduced with a T cell receptor specific for MAGE-C2/HLA-A2 vector, referred to as “antigen-specific T cells”. The later T cells allowed studies into the role of antigen-specific activation of T cells, to which end SUM159PT cells were used as these cells express the cognate antigen (MAGE-C2/HLA-A2). Both types of T cells were cultured in RPMI media, supplemented with 10% fetal bovine serum and IL-2 (360 IU/mL, Chiron, Amsterdam, The Netherlands), and Penicillin/Streptomycin. The T cell transduction process, validation of TCR expression by flow cytometry and particular culture conditions by allogeneic feeder cells are described in [24, 55].

#### Construction of the in vitro BBB assays

To develop a BBB model, HUVECs were co-cultured with human astrocytes on opposite sides of a transwell insert [13]. Twenty-four-wells transwell polycarbonate inserts (surface area 0.33 cm^2^, pore size 3 µm, Becton Drive, Franklin Lakes, NJ, USA) were coated with 2% gelatin (Sigma) for 45 min. The transwell inserts were placed upside-down and ~ 100,000 human astrocytes/inserts were seeded at the bottom side of the inserts. The cells were allowed to adhere for 3 h at 37 °C in a 5% CO_2_ incubator and were fed every 15–30 min. After 3 h, inserts were flipped and placed in 24-well plates. 1 mL of astrocyte media was added to the lower chamber and astrocytes were allowed to grow for 1 day. Fifty-thousand endothelial cells were plated on the upper chamber of the inserts and the cultures were placed in the incubator for 3 days. The permeability of the BBB model was verified by adding trypan blue dye to the upper chamber and incubating the model for 30 min at 37 °C. Medium from the lower chamber was collected and absorbance was measured at 595 nm. The permeability of the BBB model by trypan blue was included in duplicate in each experiment.

#### In vitro BBB-T cell response functional studies

To investigate the influence of T lymphocytes on the ability of tumor cells to cross the BBB, MDA-MB-231, MDA-MB-231-BM and SUM159PT breast cancer cells were co-cultured with T cells (no specific binding occurred between T cells and breast cancer cells). In addition, SUM159PT breast cancer cells were co-cultured with antigen-specific T cells (specific binding occurred between T cells and breast cancer cells). The optimal ratio of tumor cells and T cells was achieved following a titration to reach the best balance between cell killing and migration. The co-culture ratio of breast cancer cells over T cells was 3:1. After co-culturing for 3 days, T cells were removed by three washes using PBS. The breast cancer cells were harvested and labelled with 5 µM CFMDA cell tracker green (Invitrogen) for 45 min in serum-free medium. The breast cancer cells were collected and re-suspended in RPMI medium supplemented with l-glutamine, 10% fetal bovine serum and Penicillin/Streptomycin. Ten thousand cells were seeded in the upper chamber of the BBB model and incubated overnight at 37 °C. The cells which had passed through were recorded by confocal microscopy of the lower chamber after removing the transwell inserts.

To investigate whether the cytokines secreted by T cells are responsible for the changes of breast cancer cells, cells of the three breast cancer cell lines were incubated with 5 ml conditioned media collected from T cells for 3 days. Then breast cancer cells were washed, harvested and labelled using the same method as described above. Ten-thousand cells were seeded on the upper chamber of the insert and incubated overnight at 37 °C. As a control, ten-thousand breast cancer cells of the three breast cancer cell lines, without exposure to either T cells or to conditioned media from T cells, were used. These experiments were repeated 10 times. As a negative control, fibroblasts isolated from non-cancerous tissue were used.

To study the effect of IL-2 (which is essential in culturing T cells) and IFN-γ (a cytokine produced by T cells) the three types of breast cancer cell lines were incubated with IL-2 (360 IU/ml) and with IFN-γ (10 and 20 µg, Bio-Connect, Huissen, The Netherlands) separately for 3 days. Subsequently, breast cancer cells were washed, labelled with cell tracker green and seeded in the upper chamber of the BBB model. These experiments were repeated three times. Moreover, to study the effects of T cells, conditioned media of T cells, IL-2 and IFN-γ, on the permeability of the BBB model, they were added to the upper chamber of the BBB model and incubated overnight. Subsequently, the permeability of the BBB model was investigated by adding trypan-blue dye (20% in RPMI media) to the upper chamber and incubated for 30 min at 37 °C. Medium from the lower chamber was collected and absorbance was measured at 595 nm. In addition, the effect of the mentioned factors on the permeability of the BBB model was checked by seeding breast cancer cells in the upper chamber of the BBB model overnight. These experiments were repeated three times.

#### Confocal laser-scanning microscopy and Quantification of the migrated cells

Confocal images were obtained using a Zeiss LSM510 confocal laser-scanning microscope equipped with a 488 nm Argon-laser and a Plan-Neofluar 20× objective with NA 0.5 (Zeiss, Oberkochen, Germany). Images were made with a pixelsize of 0.9 µm. Pictures were submitted to ImageJ software version 1.49S (http://www.fiji.sc) and used to calculate the number of cells per mm^2^.

#### Proteomics measurements

MDA-MB-231 and MDA-MB-231-BM breast cancer cells were co-cultured with T cells in 3: 1 ratio, and SUM159PT was co-cultured with antigen-specific T cells for 3 days (following the same method described earlier). As controls, MDA-MB-231, MDA-MB-231-BM and SUM159PT cells were cultured without T cells for 3 days. All cell cultures were washed three times with PBS to remove T cells before they were scraped and collected in 1.5 mL Eppendorf tubes. Three additional washing steps with PBS were applied. After removing the supernatant, the cell pellets were immediately snap-frozen on dry-ice and stored in − 80 °C until the time of preparation. After thawing the samples, they were prepared and measured on nano LC as described previously [35].

#### Proteomics data analysis

From the raw data files of the Orbitrap Fusion mass spectrometer, MS/MS spectra were extracted and converted into mgf files using MSConvert of ProteoWizard1 (version 3.0.06245). All mgf files were analyzed using Mascot (version 2.3.02; the Matrix Science, London, UK), which was used to perform searches against the Uniprot_sprot_2014_09 database; Homo sapiens species restriction; 66,244 sequences. For the database search the following settings were used: a maximum of two miss cleavages, oxidation as a variable modification of methionine, carbamidomethylation as a fixed modification of cysteine and trypsin was set as enzyme. A peptide mass tolerance of 10 ppm and a fragment mass tolerance of 0.5 Da were allowed. An ion score of 40 was used as cut-off value.

Scaffold software (version 4.4.3, Portland, OR) was used to summarize and filter MS/MS based peptides and protein identifications. Protein identifications were accepted if they could be established at greater than 99.0% probability and contained at least two identified peptides. Proteins that contained similar peptides and could not be differentiated based on MS/MS analysis alone were grouped. Using these criteria, Scaffold generated a list of identified proteins (Minimum: 6% coverage and 2 peptides), including the number of sequenced peptides that corresponded to these proteins. The identified proteins of breast cancer cells (of the three cell lines) that were co-cultured with T cells were compared to those of the breast cancer cells cultured without the T cells. The comparison was done based on 2 sample *t* test, and the *P* values of all proteins were calculated and corrected for multiple variants using Benjamini–Hochberg. A protein was considered as a differentially expressed protein if *P* < 0.05, and all three breast cancer cell lines in one group showed the same direction of expression.

The mass spectrometry proteomics data have been deposited to the ProteomeXchange Consortium via the PRIDE [60] partner repository with the dataset identifier PXD006750.

#### *GBP1* silencing

To study the role of *GBP1* in crossing the BBB by the tumor cells, silencing transfection experiments were performed. A mix of four siRNA sequences that target *GBP1* mRNA and another mix of non-targeting siRNA (referred to as siSham) were obtained from Dharmacon (GE health care, The Netherlands). Silencing experiments were performed using transfection buffer 1, following the manufacturer’s instructions (Dharmacon, GE health care, The Netherlands). MDA-MB-231 and MDA-MB-231-BM breast cancer cell lines were transfected for 24 h. RNA was isolated using RNeasy Micro Kit (Qiagen), and the silencing efficiency was evaluated by reverse transcriptase quantitative PCR (RT-qPCR) using TaqMan gene expression assay (Applied Biosystems).

When siGBP1 proved to be efficient, the transfection experiments were performed for 24 h, then breast cancer cells were co-cultured with T cells (3:1 ratio) for an additional 24 h. The co-culturing time was reduced to 24 h, well within the effective period of siGBP1. Afterward, T cells were washed away with PBS, and breast cancer cells were harvested and labelled with green tracker as described above. Ten-thousand cells were seeded on the upper chamber of the BBB model as described above.

##### Immunohistochemistry

Formalin fixed, paraffin embedded (FFPE) tissue samples that morphologically matched the primary FF tissues were used to perform immunohistochemistry. We retrieved 11 FFPE samples of the 22 FF samples that were used for discovery. The T cell markers were stained with an automated IHC staining system (Ventana BenchMark ULTRA; Ventana Medical System Inc. Tucson, AZ). CD3 (0.4 µg/mL, clone 2GV6, Ventana, Tuscan, AZ) antibody was used as general marker for T cells; CD4 (2.5 µg/mL, clone PS35, Ventana, Tuscan, AZ) and CD8 (1:100 dilution, clone C8/144B, Dako, Heverlee, Belgium) were used as markers for T helpers and T cytotoxic subsets, respectively. The staining was performed according to the manufacture instructions. In addition, GBP1 (1:250 dilution, Santa Cruz Biotechnology, Heidelberg Germany) was used according to the manufacture instructions. In addition to the 11 FFPE samples of the discovery set, we used 20 independent samples for extra validation. The clinical information of the samples is summarized in Table 1.

### In vivo mouse model

#### T cell isolation and tissue culture

T cells were obtained from the spleen of 4–6-week-old FVB mice weeks. Splenocytes were sorted using anti-mouse CD4-Pe (BD Pharmigen catalog. 553048) and activated with anti-mouse CD3e clone 145-2C11 (BD Pharmigen catalog. 553066) coated plates, soluble anti-mouse CD28 (37.51) (Tonbo Biosciences catalog. 70-0281-U500), and mouse IL-2 (Miltenyi Biotec catalog. 130-094-054). The ErbB2-P cell line was established from MMTV driven-NeuNT transgenic mammary tumors in mice [34]. They express Luciferase and gfp. ErbB2-P are cultured in vitro in DME media supplemented with 10% fetal bovine serum (FBS), 2 mM l-Glutamine, 100 IU/ml penicillin/streptomycin, and 1 mg/ml amphotericin B. The ErbB2-P was co-cultured with T cells in RPMI medium supplemented with 10% FBS and Penicillin–Streptomycin for 3 days and then sorted out and injected in syngeneic animals into the heart. 3 weeks later, metastasis burden was analyzed by bioluminescence imaging (BLI).

#### qRT-PCR

RNA (QIAGEN) from sorted gfp + ErbB2-P cancer cells was used to generate cDNA (iScript cDNA Synthesis Kit Bio-Rad catalog. 1708890). Gene expression was analyzed using SYBR green gene expression assays (GoTaq^®^ qPCR Master Mix Promega catalog. A6002). Primers: *Gbp1* (Sequence of the primer 5–3): 5′-3′GGGCAGCTGTCTTTGGGTAGAC, 3′-5′AGCATGAGGCCCTAGGAGCTGT. Quantitative PCR reaction was performed on QuantStudio 6 Flex Real-Time PCR System (Applied Biosystems) and analyzed using the software QuantStudio 6 and 7 Flex Software.

#### Flow cytometry

ErbB2-P and T cells were isolated from the co-culture based on gfp expression using FACS ARIA Ilu sorter.

#### Animal experiments

All animal experiments were done in accordance with a protocol approved by the CNIO, Instituto de Salud Carlos III and Comunidad de Madrid Institutional Animal Care and Use Committee. ErbB2-P cells were injected intracardiacally. Briefly, a cell suspension containing 10^5^ ErbB2-P cells in a volume of 100 μl was injected in the left cardiac ventricle of anesthetized 4–6-week-old FVB mice. Tumor burden was evaluated by bioluminescence imaging using the IVIS-200 imaging system from Xenogen as previously described. Metastases were defined with BLI as those with signal equal or above 2x10^3^ photon flux.

#### Statistics

*P* value is calculated using two-tailed *t* test.

#### Free-floating immunofluorescence

Tissue for immunofluorescence was obtained after overnight fixation with PFA 4% at 4 °C. Slicing of the brain was done using a sliding microtome (Fisher). Brain slices (80 μm) were blocked in NGS 10%, BSA 2%, Triton 0.25% in PBS for 2 h at room temperature (RT). Primary antibodies were incubated overnight at 4C in the blocking solution and the following day for 30 min at RT. After extensive washing in PBS-Triton 0.25%, the secondary antibody was added in the blocking solution and incubated for 2 h. After extensive washing in PBS-Triton 0.25%, nuclei were stained with Bis-Benzamide for 7 min at RT. Primary antibody GFP (Aves Labs, ref. GFP-1020, 1:1000). Secondary antibody is Alexa-Fluor anti-chicken 488 (Invitrogen).

#### Quantification of brain metastases histology

80 um sections were obtained from each brain generating 10 series. One series containing 10–12 slices representative of the whole organ was used for immunofluorescence analysis. The staining of gbp1 was performed and positive lesions were quantified under a fluorescence microscope. Total number of metastases per series was obtained and plotted.

## Results

### Breast cancers associated with brain metastasis express genes involved in the T lymphocyte response

**Fig. 1:**
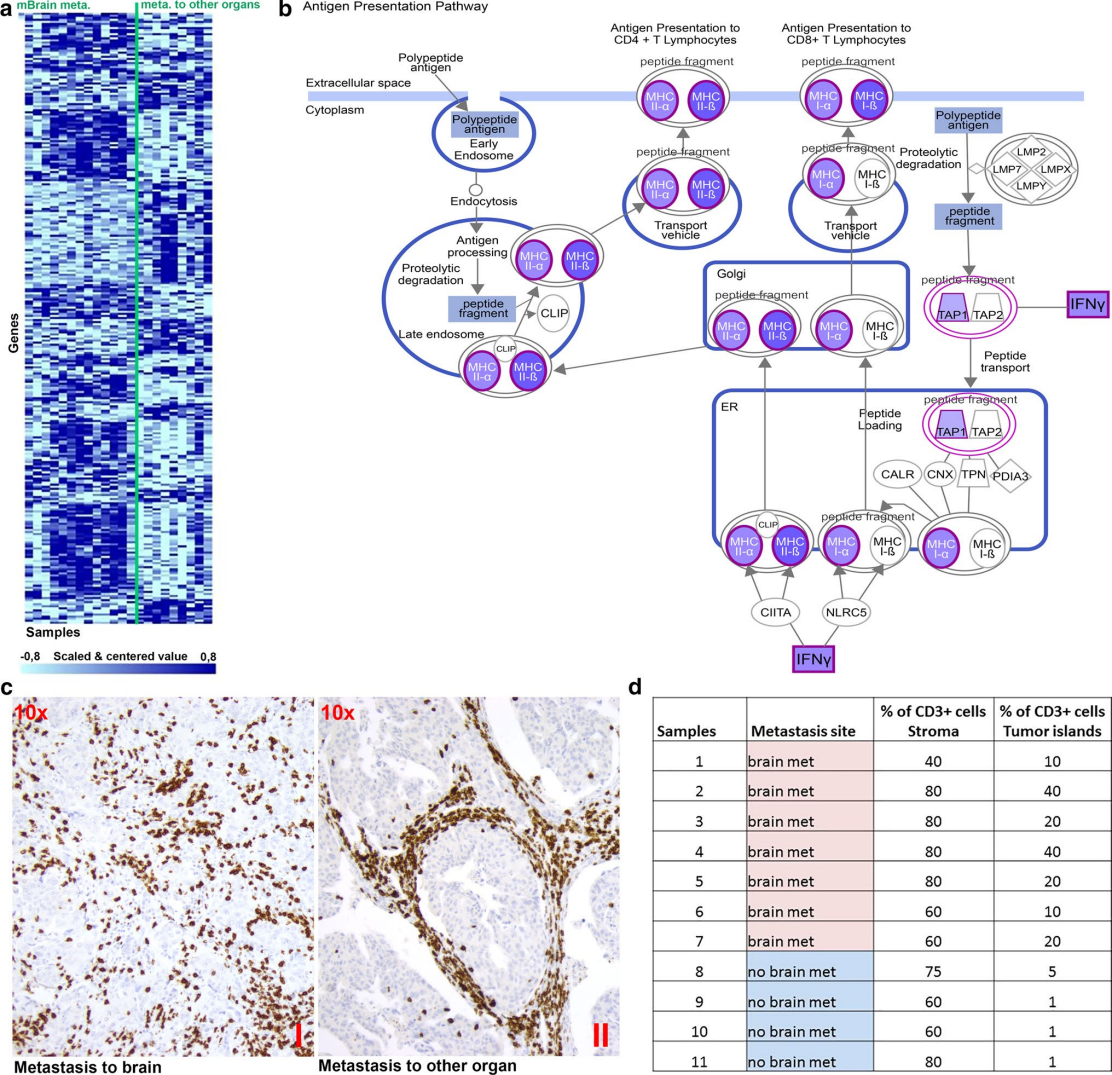
Breast cancer samples associated with brain metastasis express genes involved in the T lymphocyte response. **a** Heat map of the 298 differentially expressed probes between the primary breast cancer samples that developed metastases, with or without brain metastasis. **b** Pathway analysis revealed the involvement of the T cells response in the formation of brain metastasis. In this “Antigen Presenting Pathway”, the up-regulated genes in the samples that developed brain metastasis are shown in purple. **c** (1) In the primary breast cancer samples associated with brain metastasis, T cells invade the tumor tissue. (2) In the samples associated with metastasis to other sites, the T cells remain in the tumor stroma. (CD3, 100×). **d** Percentages of TILs in the primary breast cancer samples of patients who developed metastasis to brain or to other organs

Comparing the gene expression profiles of primary breast cancer samples that developed brain metastasis with those that developed metastasis to other organs resulted in 298 differentially regulated genes at *P* < 0.05 (Fig. 1a). Among the significant genes, 176 were up-regulated in the group of tumors associated with brain metastasis, while 122 genes were up-regulated in the group associated with metastasis to other organs. To prioritize these genes, we ran functional and pathways analyses. The function annotation tool DAVID revealed that “regulation of T cell activation” was most prominent among the samples associated with brain metastasis (*P* value < 0.00002; enrichment score of 6.02). Pathway analysis pointed to a prominent involvement of the “T lymphocyte response” based on both methods (Ingenuity and Global testing) (Tables 2, 3). Moreover, all involved genes in the pathway were up-regulated in the group associated with the brain metastases (Fig. 1b, Table 2).

**Table 2:**
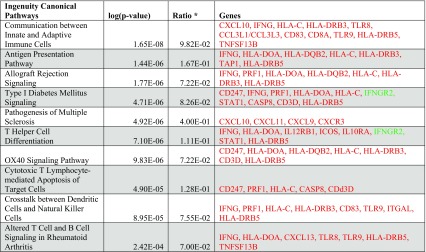
Top ten canonical pathways (ingenuity)

Red: up-regulated, and green: down-regulated, in primary breast cancers samples associated with metastasis to brain

*Ratio of genes found over their total number in a particular pathway

**Table 3:** Top ten canonical pathways resulted from global testing

Global test canonical pathways	Comparative *P* value^*^	*P* value
The 41BB-dependent immune response	0.004	0.0016
Chaperones modulate interferon signaling pathway	0.004	0.0043
Th1–Th2 differentiation	0.008	0.0045
Roles of arrest independent recruitment of Src Kinases in GPCR signaling	0.012	0.0055
X arrest ins in GPCR desensitization	0.012	0.0101
B lymphocyte cell surface molecules	0.011	0.0117
Role of Tob in T cell activation	0.006	0.0118
Dendritic cells in regulating TH1 and TH2 development	0.009	0.0144
Activation of Csk by cAMP dependent protein kinase inhibits signaling through the T cell receptor	0.012	0.0180
NO2-dependent IL-12 pathway in NK cells	0.015	0.0187

*P value calculated by 1000 re-samplings

We screened 11 tumors for the presence of tumor infiltrating lymphocytes (TILs) according to the criteria of the TILs working group 2014 [48], and found that TILs were confined to the stroma surrounding tumor islands in the primary breast cancer samples of patients who had developed metastasis to other organs (Fig. 1c, d). In contrast, TILs had invaded the tumor island in patients who had developed cerebral metastases. This observation suggested that T cells may play a role in increasing the ability of breast cancer patients to develop brain metastasis.

### T lymphocytes increase the ability of breast cancer cells to cross an in vitro BBB model

**Fig. 2:**
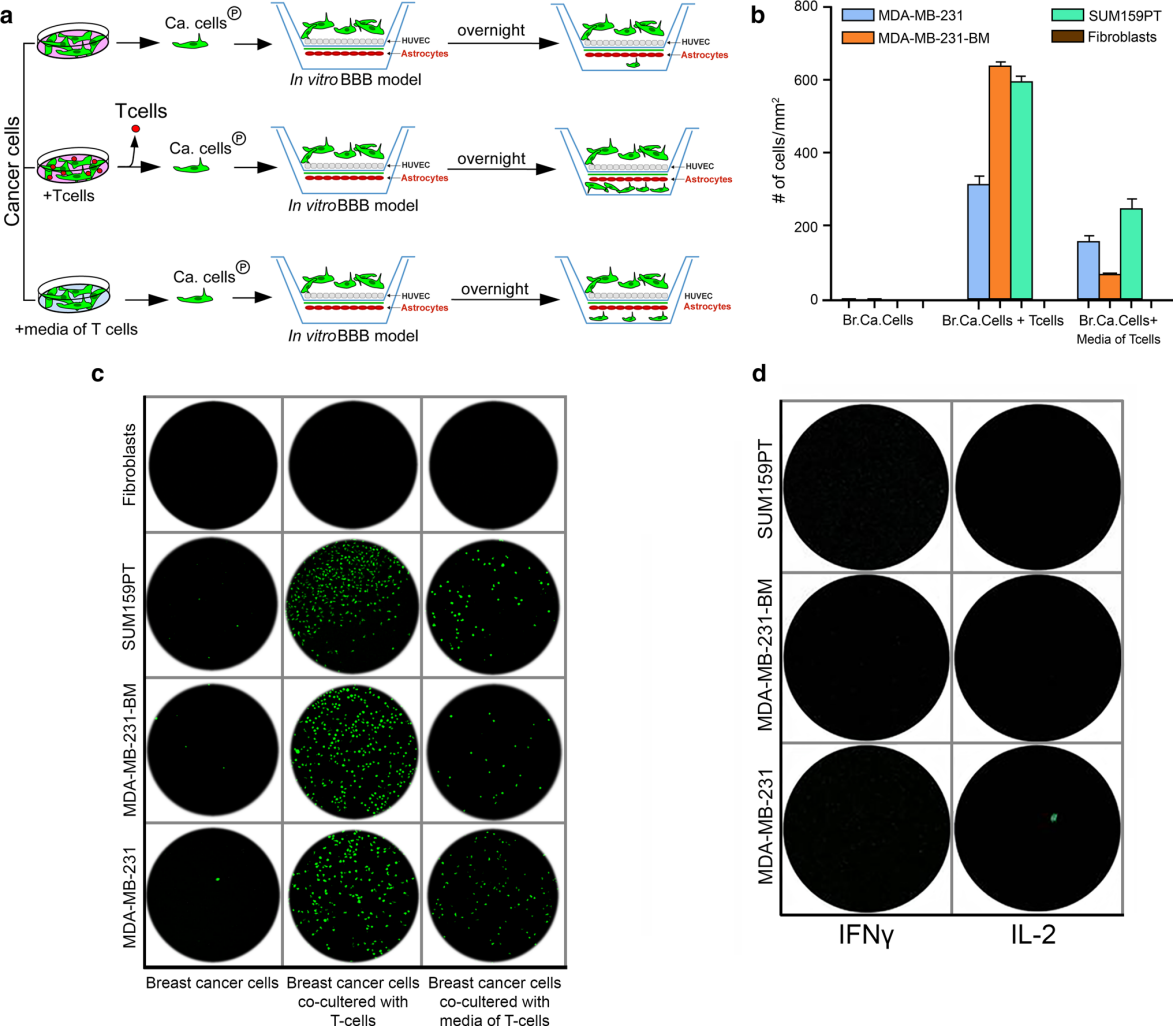
T lymphocytes increase the ability of breast cancer cells to cross the BBB in the in vitro model. **a** Schema of experimental design. **b** Three breast cancer cell lines (MDA-MB-231, MDA-MB-231-BM, and SUM159PT) showed a limited ability to cross the in vitro BBB (left column, lower three fields). Following co-culture with T cells, the ability of breast cancer cells to cross the BBB increased significantly (middle column, lower three fields). Conditioned media of T cells also increased the ability of breast cancer cells to cross the BBB, but to a lesser extent (right column, lower three fields). Neither T cells, nor their media, facilitated fibroblasts to cross the BBB (upper row). All experiments were repeated ten times. **c** Quantitative representation of B (error bars indicate standard deviation) (Br. Ca. = breast cancer). **d** IFNγ and IL-2 did not change the ability of breast cancer cells to cross the BBB. These experiments were repeated three times

To functionally validate the importance of T cells to stimulate brain metastasis, we developed an in vitro BBB model and used three breast cancer cell lines (MDA-MB-231, MDA-MB-231-BM, and SUM159PT). These cell lines are known for their ability to cross the BBB [3, 45], and they all were able to cross the BBB of our in vitro model. However, the number of cells that crossed the artificial BBB overnight was very limited (< 10 cells). We co-cultured breast cancer cells with T cells that were isolated from normal donors for 3 days, removed the T cells and added the breast cancer cells to the BBB model (Fig. 2a). The number of breast cancer cells that crossed the BBB increased significantly (> 300–650 cells) (Fig. 2b, c). To investigate if the interaction between breast cancer cells and T cells was necessary for the dramatic effect that we observed, we run two experiments. First, we incubated breast cancer cells with conditioned media from T lymphocytes (without including T cells) and found an increased ability of breast cancer cells to cross the BBB albeit less significantly (> 80–250 cells) (Fig. 2b, c). Second, we co-cultured SUM159PT breast cancer cells (that express the cognate antigen MAGE-C2/HLA-A2) with antigen-specific T cells (CD3^+^ T lymphocytes transfected with MAGE-C2/HLA-A2 vector). The specific interaction between the breast cancer cells and the T lymphocytes increased the ability of the breast cancer cells to cross the BBB significantly (> 400 cells) (Supp. Figure 1). To investigate if T cells have the same effect on normal cell, we replaced the tumor cells for normal fibroblasts. Co-culturing fibroblasts with T lymphocytes, or with conditioned media from T lymphocytes, did not change their ability to cross the BBB (Fig. 2b, c). To investigate if T lymphocytes have a direct influence on the permeability of the BBB in the model, we added T cells, antigen-specific T cells, and their conditioned media to the upper chamber of the model. T lymphocytes were able to cross the BBB, but neither the T cells, nor their conditioned media, changed the permeability of the BBB (data not shown). We also tested a possible direct effect of interferon gamma (IFNγ) secreted by the T cells, on the tumor cells, by incubating the breast cancer cells with several concentrations of IFNγ, but no effect on the ability to cross the BBB model was noticeable. In addition, we tested the effect of IL-2 (a required growth factor for culturing T lymphocytes) on tumor cells, but no facilitating effect was observed either (Fig. 2d). Both IFNγ and IL-2 did not change the permeability of the BBB (data not shown). These experiments confirmed that T lymphocytes and their secreted factors increase the ability of breast cancer cells to cross the in vitro BBB model, and also proved that antigen-specific interaction between breast cancer cells and T lymphocytes is not necessary to promote the discovered facilitating effect.

### GBP1 protein is involved in changing the ability of breast cancer cells in crossing the BBB

**Fig. 3:**
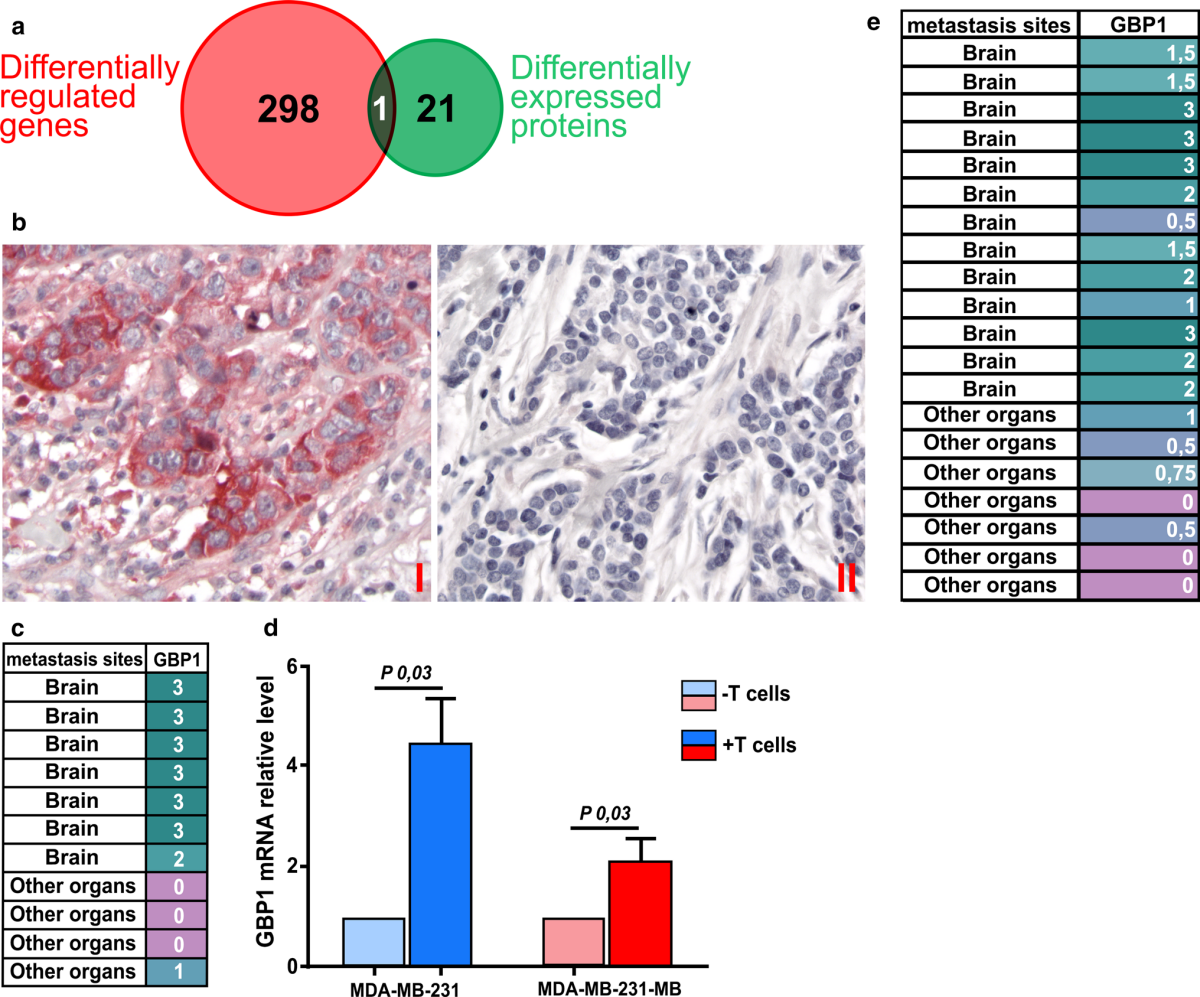
Immunohistochemistry for GBP1 in primary breast cancer samples. **a** Venn diagram illustrating the overlap of differentially regulated genes and proteins. **b** (I) Expression of GBP1 protein in primary breast cancers with brain metastasis. (II) GBP1 protein in primary breast cancers with metastases to organs other than brain. **c** Semi-quantitative results of immunohistochemistry for GBP1 using the discovery sample set. The color scale represents the scores of the immunohistochemical staining, ranging from 0 = no expression to 3 = highest expression. **d** RT-PCR results of the GBP1 expression in MDA-MB-231 and MDA-MB-231-BM before and after co-culturing with T lymphocytes. For both cell lines, the GBP1 expression before and after co-culturing with T cells differ significantly. Bars indicate mean values ± SEM, from three independent experiments. **e** Semi-quantitative results of immunohistochemistry for GBP1 using 20 independent primary breast cancer samples. The color scale represents the scores of the immunohistochemical staining, ranging from 0 = no expression to 3 = highest expression

Next, we aimed to identify the changes that occur in breast cancer cells after co-culturing with T lymphocytes responsible for their increased capacity to pass through the BBB. Therefore, we measured the proteome of breast cancer cells before and after co-culturing with T cells by mass spectrometry. The proteomics comparisons lead to the identification of 21 differentially regulated proteins between the two groups (out of over 2500) at *P* < 0.05, 12 of which were up-regulated in the cells exposed to the T cells (Tables 4, 5). The 21 differentially regulated proteins were compared to the 298 differentially expressed genes identified in the mRNA gene expression arrays (Fig. 3a) and GBP1 was the only protein that matched at the mRNA level. GBP1 protein was exclusively measured and identified in all three breast cancer cell lines that were co-cultured with T lymphocytes (*P* < 0.001), and the *GBP1* gene was up-regulated in the primary breast cancer samples that developed brain metastasis (*P* < 0.05 and 1.5 fold change). Furthermore, staining for GBP1 was positive only in the samples of the primary breast cancers of the patients who developed brain metastases, while not in the samples of patients who developed metastasis to other organs (Fig. 3b, c). Furthermore, the tumor areas with and without the expression of GBP1 were morphologically indistinguishable, except for the presence of TILs in the former. To further confirm our findings, we validated the upregulation of GBP1 after co-culturing breast cancer cells with T lymphocytes by RT-PCR (Fig. 3d). In addition, immunohistochemical staining for GBP1 of 20 independent samples of which 13 developed brain metastasis showed positivity only in the ER- breast cancer samples of patients who developed brain metastasis (Fig. 3e).

**Table 4:** Differentially expressed proteins of breast cancer cells before and after co-culturing with T cells

#	Identified proteins	Gene	Accession # (uniprot)	Molecular weight (kDa)	*T* test (*p* value)	Breast cancer cells co-cultured with T cells			Pure breast cancer cells		
						MDA-MM-231	MDA-MM-231 BM	SUM159PT	MDA-MM-231	MDA-MM-231 BM	SUM159PT
1	Rab11 family-interacting protein 5	RAB11FIP5	Q9BXF6	70	< 0.00010	2	2	2	0	0	0
2	AP-1 complex subunit gamma-1	AP1G1	O43747	91	< 0.00010	2	2	2	0	0	0
3	WD repeat-containing protein 43	WDR43	Q15061	75	< 0.00010	2	2	2	0	0	0
4	Eukaryotic translation initiation factor 4E	EIF4E	P06730	25	< 0.00010	2	2	2	0	0	0
5	NADH–cytochrome b5 reductase 1	CYB5R1	Q9UHQ9	34	< 0.00010	2	2	2	0	0	0
6	SAFB-like transcription modulator	SLTM	Q9NWH9	117	0.001	3	3	2	0	0	0
7	Guanylate-binding protein 1	GBP1	P32455	68	0.0013	3	2	3	0	0	0
8	Transmembrane emp24 domain-containing protein 2	TMED2	Q15363	23	0.0016	3	2	2	0	0	0
9	Serotransferrin	TF	P02787	77	0.0029	4	4	6	0	0	0
10	Arf-GAP domain and FG repeat-containing protein 1	AGFG1	P52594	58	0.0029	2	2	3	0	0	0
11	DnaJ homolog subfamily C member 11	DNAJC11	Q9NVH1	63	0.0029	2	2	3	0	0	0
12	60S acidic ribosomal protein P1	PLP1	P05386	12	0.019	2	2	2	0	0	0

Detected proteins in the three breast cancer cell lines after co-culturing with T cells

Numbers represent the number of unique peptides belonging to a specific identified protein in each sample

**Table 5:** Detected proteins in the three breast cancer cell lines that had not been co-culturing with T cells

#	Identified proteins	Gene	Accession # (uniprot)	Molecular weight (kDa)	*T* test (*p* value)	Breast cancer cells co-cultured with T cells			Pure breast cancer cells		
						MDA-MM-231	MDA-MM-231 BM	SUM159PT	MDA-MM-231	MDA-MM-231 BM	SUM159PT
1	Charged multivesicular body protein 4b	CHMP4B	Q9H444	25	< 0.00010	0	0	0	2	2	2
2	Sjoegren syndrome/scleroderma autoantigen 1	SSSCA1	O60232	21	0.00098	0	0	0	3	3	2
3	C-terminal-binding protein 2	CTBP2	P56545	49	0.0015	0	0	0	2	3	2
4	Programmed cell death protein 6	PDCD6	O75340	22	0.0018	0	0	0	3	2	3
5	Transcription elongation factor A protein-like 3	TCEAL3	Q969E4	23	0.0023	0	0	0	3	2	2
6	UBX domain-containing protein 1	UBXN1	Q04323	33	0.003	0	0	0	2	2	3
7	ER membrane protein complex subunit 2	EMC2	Q15006	35	0.003	0	0	0	2	2	2
8	U4/U6 small nuclear ribonucleoprotein Prp31	PRPF31	Q8WWY3	55	0.0051	0	0	0	3	4	2
9	ATPase ASNA1	ASNA1	O43681	39	0.016	0	0	0	4	2	2

**Fig. 4:**
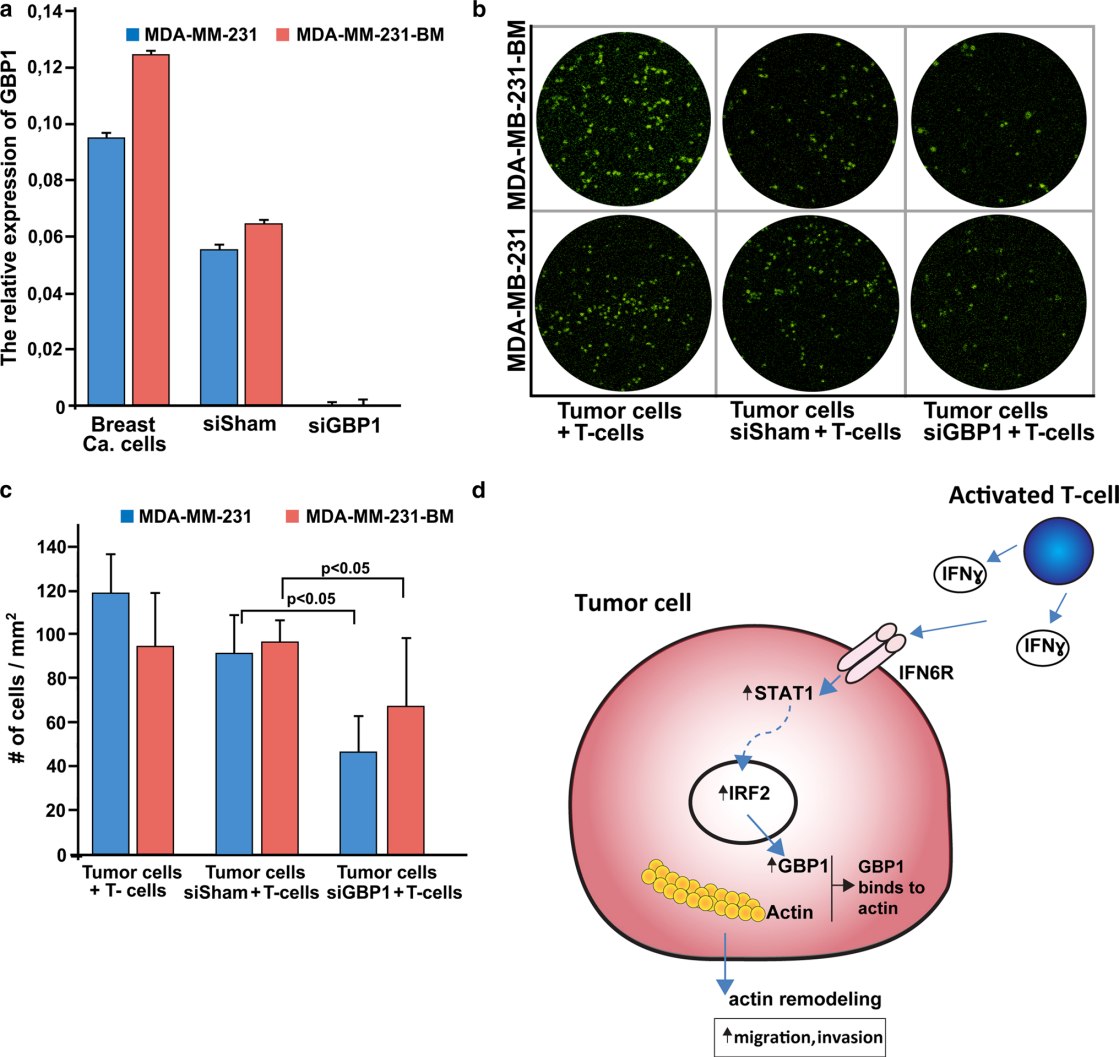
GBP1 affects crossing of breast cancer cells through the BBB. **a** RT-PCR results of *GBP1* expression showing successful silencing. The expression of *GBP1* in breast cancer cells was compared to that of the non-targeting siRNA (siSham) (*n* = 3; bars indicate standard deviation). These experiments were repeated twice, and the results were reproducible. **b** Breast cancer cells silenced for *GBP1* and co-cultured with T cells show a reduction in their ability to pass the BBB (right column) as compared to the breast cancer cells that were silenced for siSham (middle column). As a control, breast cancer cells not silenced for *GBP1*, were co-cultured with T cells, showed their ability to cross the BBB (left column). These experiments were repeated twice, and the results were reproducible. **c** Quantitative results of B; error bars indicate standard deviation). The number of breast cancer cells that were able to cross the BBB had decreased significantly after silencing *GBP1*. **d** Cartoon illustrating the function of GBP1

The prominent effect of *GBP1* on the capacity of tumor cells to cross the modelled BBB was examined by silencing experiments. *GBP1* was silenced in MDA-MB-231 and in MDA-MB-231-BM breast cancer cell lines using pooled probes against *GBP1.* However, the expression of *GBP1* was not affected when using siSham (negative control) (Fig. 4a). Subsequently, the cancer cells were co-cultured with T cells. The silenced *GBP1* breast cancer cells showed a significant decrease in the ability to cross the BBB following co-cultured with T cells as compared to siSham cells, or to breast cancer cells that were not affected by silencing. A 30–70 fold decline in crossing of the BBB was reached following silencing of *GBP1* (Fig. 4b, c).

### T lymphocytes increase the ability of breast cancer cells to induce brain metastases in a mouse model

**Fig. 5:**
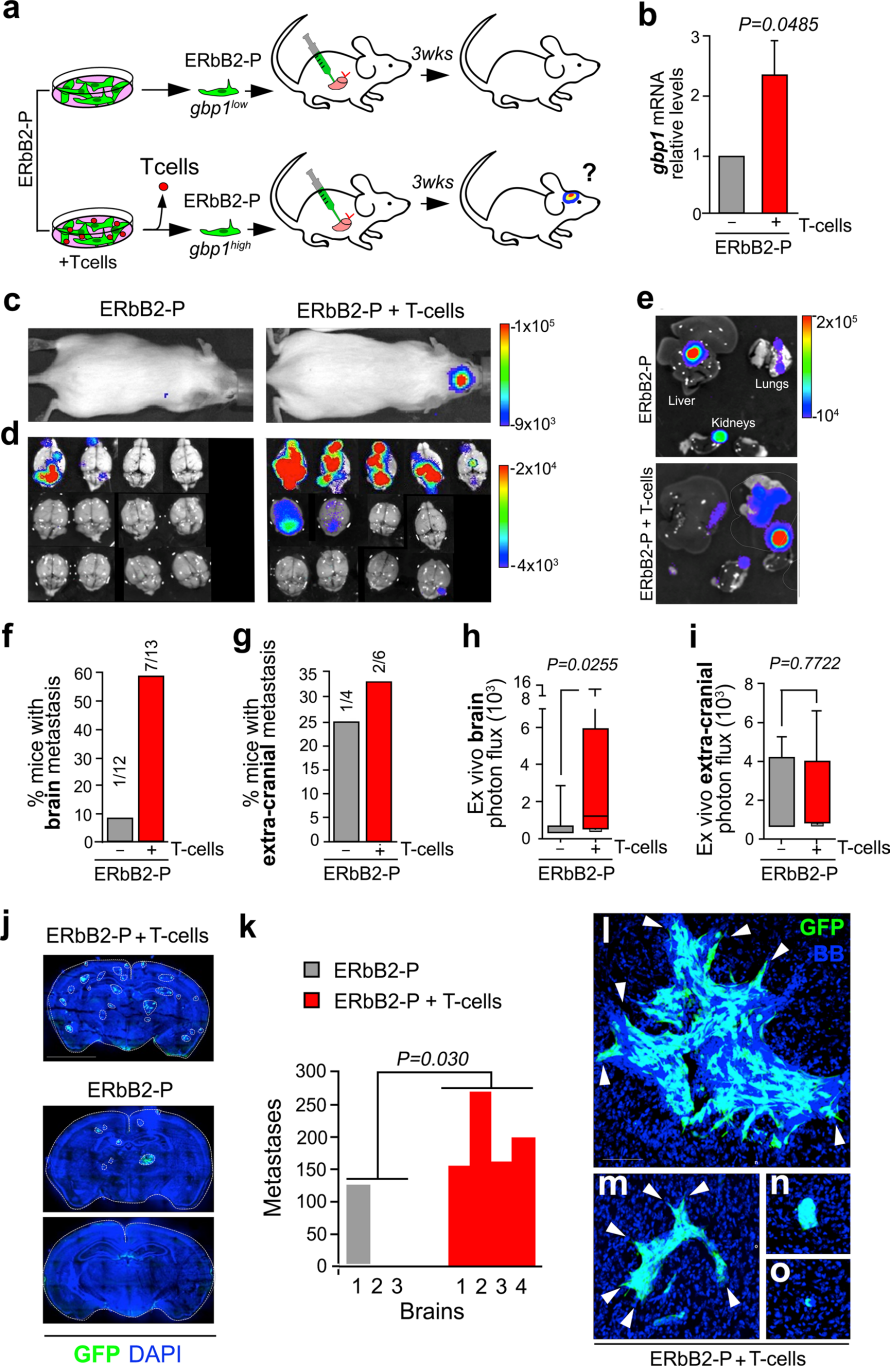
T lymphocytes facilitate brain metastasis of breast cancer in mice. **a** Schema of experimental design. **b** qRT-PCR of sorted ErbB2-P cells after being co-cultured with T cells. Values indicate mean values ± SEM, from three independent experiments. **c** Representative bioluminescence images (BLI) from mice 21 days after intracardiac injection. **d** Ex vivo BLI of brains from injected animals. **e** Representative BLI of extra-cranial metastases. **f** Graph showing the percentage of mice affected with brain metastasis. Numbers in bars indicate the absolute values g. Graph showing the percentage of mice affected with extra-cranial metastases. Numbers in bars indicate the absolute values. **g** Quantification of bioluminescence signal emitted by brains ex vivo. Error bars, minimum and maximum values reached by brains. Line in each bar indicates mean BLI value. (*n* = 12 brains, ErbB2-P; 13 brains, ErbB2-P previously co-cultured with T cells (ErbB2-P + T cells), from 2 independent experiments. *P* value is calculated using two-tailed *t* test. **h** Quantification of bioluminescence signal emitted by extra-cranial metastases (liver, lungs and kidneys) ex vivo. Error bars, minimum and maximum values reached. Line in each bar indicates mean BLI value. (*n* = 4 mice, ErbB2-P; 6 mice, ErbB2-P + T cells, from one experiment. *P* value is calculated using two-tailed *t* test. **i** Confocal scans of representative slices from different brains. Two brains from mice previously inoculated with ErbB2-P are shown to illustrate the limited seeding (upper panel) and complete absence (lower panel) of metastatic cells. Scale bar 300 mm. White line demarcates brain slices. Dotted white line surrounds gfp + metastases. **j** Quantification of mean number of metastases in representative brains from each condition. Individual brains are plotted in each experimental condition (*n* = 3 brains from ErbB2-P injected mice; 4 brains from ErbB2-P + T cells injected mice). *P* value is calculated using two-tailed *t* test. **k** L–O. Representative images to show the heterogeneity present in brains from mice injected with ErbB2-P + T cells. (l-m) Large and medium size metastases with invasive fronts (arrows) co-exist with less abundant (n) well-circumscribed metastases and (o) abundant single cell events. Scale bar 75 μm

To functionally test the ability of T cells to promote brain metastasis, we used a cancer cell line (ErbB2-P) established from a spontaneous ErbB2 + mammary tumor derived from MMTV driven-NeuNT transgenic mice [34]. This cell line does not have the ability to target the brain when injected in the systemic circulation [58]. The ErbB2-P cells were co-cultured with T cells, which were previously activated in vitro (Fig. 5a). Sorted cancer cells were initially interrogated by qRT-PCR to analyze *Gbp1* levels. Analysis of three independent experiments showed that co-culture of ErbB2-P with activated T cells induces a significant increase in *Gbp1* expression levels in the cancer cells (Fig. 5b). To interrogate the influence of T cells on metastasis, ErbB2-P cells were intra-cardially inoculated in immunocompetent syngeneic mice (Fig. 5a). 21 days after inoculation, most of the clones of the ErbB2-P cell line were not able to generate brain metastasis with only one animal out of 12 (8.3%) showing bioluminescence in the brain (Fig. 5c, d, f, h). This limited potential to grow in the brain of the parental cell line dramatically increased when cancer cells inoculated had been previously in contact with T cells. Seven out of thirteen (53.8%) animals inoculated with ErbB2-P co-cultured with T cells developed brain metastasis (Fig. 5c, d, f, h). This sixfold increase in the ability to generate secondary tumors in the brain was not mimicked in other organs since ex vivo analysis of lungs, liver, kidneys, adrenal glands, and bones (data not shown) did not show any difference nor in the percentage of animals affected (ErbB2-P: 25% mice with extra-cranial disease; ErbB2-P + T cells: 33.3%) neither in the bioluminescence signal from ex vivo analysis (Fig. 5e, g, i). We conclude that activated T cells are sufficient to increase the ability of breast cancer cells to develop brain metastasis. To investigate the brain metastatic phenotype in more detail, in particular if there was increased access of cancer cell clones in the brain parenchyma, or if there is also an increased ability to colonize the brain, we microscopically examined the brains. Most brains inoculated with ErbB2-P cells did not show any cancer cell, even at the single cell level with the exception of the BLI + one (Fig. 5j, k). The ErbB2-P cells that were co-cultured with T cells consistently showed multiple cancer cell clones within the perivascular spaces (indicative of the ability to get access to the brain parenchyma; Fig. 5j, k), suggesting an increased ability to cross the BBB. In addition, increased size and invasive fronts at the metastatic deposits was observed, indicative of increased capability of parenchyma invasion (Fig. 5l, m, in comparison with Fig. 5n, o). This observation suggests an additional influence of T cells providing cancer cells with further capabilities to advance from the first extravasation compartment. We conclude that the influence of the T cells extends beyond making the breast cancer cells pass the BBB, but also makes them invade the brain tissue.

## Discussion

The identification of the mechanisms and underlying molecular pathways that cancer cells use to cross the BBB is important for the development of strategies to prevent cerebral dissemination. In this study, we found the T lymphocyte response most prominently involved in the formation of cerebral metastases of ER- breast cancer patients. The involvement of T lymphocytes in the metastatic potential of breast cancer has been noticed previously, particularly implicating induction of immune tolerance by regulatory T lymphocytes [2, 5, 33, 39]. The present results, however, reveal an entirely different effect of T cells, namely, that T lymphocytes and their secreted factors change the expressional profiles of tumor cells, thereby increasing their ability to cross the BBB. It is known that the immune system mediates primary tumors in their proliferation and invasion by secretion of inflammatory cytokines, chemokines, autoantibodies, proteases, and more. The role of the immune system in the formation of metastases is complex and far from understood [1, 8, 28, 31]. To some extent, the present findings are reminiscent of the T cell involvement inducing receptor activator of nuclear factor-κB (RANK) signaling causing pulmonary metastases in a mouse breast cancer model [56]. However, the results obtained using human breast cancer samples show that the presence of T cells correlates with the formation of cerebral metastasis. This finding has not been reported previously.

The discovery of T cell involvement in the formation of cerebral metastasis was based on a relatively small series of samples of breast cancer patients. Therefore, we confirmed our findings at the functional level using in vivo and in vitro models. Injecting mice with breast cancer cells that were co-cultured with T cells proved that T cells play an important role in increasing the ability of breast cancer cells to cross the BBB and to develop brain metastasis. Interestingly, co-culturing breast cancer and T cells did not increase the tendency to metastasis to organs other than brain. The in vitro BBB model we used is composed of human endothelial cells and astrocytes, closely reflecting the normal barrier function of human BBB. The ability of breast cancer cells to cross the BBB improved significantly when the tumor cells were co-cultured with T cells. The facilitating effect was observed after co-culturing with T cells isolated from normal donors as well as with antigen-specific T cells, indicative of antigen independency. Incubation of the breast cancer cells with the conditioned media of the T cells showed similar results, pointing to the importance of particular factors secreted by T lymphocytes. In an effort to identify the factors that caused the facilitating effect, we incubated breast cancer cell with IFNγ that is secreted almost exclusively by T cells. Interestingly, IFNγ did not change the ability of breast cancer cells to cross the BBB, nor did it change the permeability of the BBB itself. Similar results were obtained when incubating breast cancer cells with IL-2. It could be argued that instead of single proteins, several secreted proteins and cytokines are necessary to induce the facilitating effect. The complex interplay between T cells and the humoral immune system was demonstrated in the MMTV-PyMT mouse model, where IL-4-expressing CD4 + T lymphocytes indirectly promote invasion and subsequent metastasis of mammary adenocarcinomas [8]. In another mouse breast cancer model, the effects of interleukin (IL)-1β on the IL-17 expression of gamma delta (γδ) T cells were shown, affecting neutrophils and suppression of CD8 + T cells, also leading to the formation of metastases [7].

Among the 21 differentially expressed proteins in the three breast cancer cell lines following co-culturing with T cells, only the *GBP1* gene was found to be overexpressed in the set of primary breast cancer samples that developed brain metastasis. RT-PCR results confirmed that GBP1 expression is significantly upregulated in breast cancer cells after co-culturing with T lymphocytes. Further confirmation of the expression of GBP1 was confirmed by immunohistochemistry in the discovery sample set and in an additional 20 independent samples. GBP1 positive cells were detected in tumor areas, where T lymphocytes invaded from the stroma in between the tumor cells. Human GBP1 is a secreted GTPase that is induced by IFNγ and mediate the antibacterial and antiviral activities of IFNγ [41]. The GBP1 protein binds to actin and plays a role in the remodeling of the fibrous actin structure thereby influencing cellular motility [14, 23, 41]. The regulation of the cytoskeleton and the remodeling of actin by GBP1 is of a great relevance in process like migration, invasion, proliferation and defense against barrier function, a possible link with the increased passage through the BBB [22, 41, 46]. However, the relation with brain invasion seems more complex since GBP1-mediated actin remodeling also contributes to the regulation of the innate and adaptive immune defense [41]. Moreover, mutations in the *GBP1* gene are among those related to the tumorigenesis of breast cancer [20] and the aggressive hormone-negative inflammatory subtype in particular [25]. In vitro studies revealed a role of *GBP1* in tamoxifen resistance [12]. The GBP1 protein is also involved in resistance to docetaxelis of prostate cancer [10], and in a recent study, it was shown that it is one of the key molecules contributing to cancer radioresistance [15]. With respect to tumors other than breast cancer, *GBP1* is considered to act as tumor suppressor gene in colorectal cancer [4], and as an effector of EGFR-driven tumor cell invasion in glioblastomas [27]. In addition, GBP1 was found to promote lymph node metastasis in esophageal squamosal cell carcinoma [26]. So far, GBP1 was not associated with brain metastasis of breast cancer. However, a recent study showed that the over-expression of GBP1 protein among others was associated with metastasis in TNBC [42]. Obviously, its expression sites, specific action, and possible partners involved in brain metastasis need further exploration. Most importantly, investigations in the effects of blocking its expression in vivo are needed to develop therapeutic strategies in preventing metastases to brain.

Our results highlight the importance of T lymphocytes and their secreted cytokines for the formation of brain metastasis originating from ER- breast cancers. This is new to current knowledge of the complex interplay between T lymphocytes and cancer cells. T lymphocytes change the expressional repertoire of breast cancer cells that promotes their ability to cross the BBB. At this point, subsequent studies are necessary to detail the role of any specific T cell subset in facilitating the breast cancers to cross the BBB. The up-regulation of the *GBP1* gene and the over-expression of GBP1 protein seem to be crucial to this effect. The predictive value of this protein of the rise of cerebral metastases should be evaluated in prospective settings.

## Electronic supplementary material

Below is the link to the electronic supplementary material.

Supplementary material 1 (DOCX 13 kb)

Supplementary material 2 (TIFF 3705 kb). Supplementary Fig. 1: T cells facilitate breast cancer cells to cross the BBB in an antigen-independent fashion. a SUM159PT breast cancer cell line that express the cognate antigen MAGE-C2/HLA-A2 was co-cultured with antigen-specific T cells (CD3^+^ T lymphocytes transfected with MAGE-C2/HLA-A2 vector). A similar facilitating effect was observed. b Quantitative results of A. Error bars indicate standard deviation
