# Supplementary material for: T lymphocytes facilitate brain metastasis of breast cancer by inducing Guanylate-Binding Protein 1 expression
Source: Acta Neuropathol. 2018 Jan 19;135(4):581–99. doi: 10.1007/s00401-018-1806-2 (PMC5978929; doi:10.1007/s00401-018-1806-2)
Supplement: Supplementary file 1 — Supplementary material 1 (DOCX 13 kb) [file 401_2018_1806_MOESM1_ESM.docx]

**Supplementary Table 1:**

**The characterization of the cell lines**

| **Cell lines** | **Classification** | **ER** | **PR** | **HER2** | **Invasiveness** | **Down- regulation** | **Up- regulation** | **Mutations** |
| --- | --- | --- | --- | --- | --- | --- | --- | --- |
| **MDA-MB-231 [**[**19**](#_ENREF_19)**]** | Basal-type | neg | neg | neg | High | Claudin-3  Claudin-4 E-cadherin | CD44+  EGFR | KRAS  BRAF  P53 |
| **MDA-MB-231-BM** | Basal-type | neg | neg | neg | High | Claudin-3  Claudin-4  E-cadherin | CD44+  EGFR | KRAS  BRAF  P53 |
| **SUM159PT [**[**19**](#_ENREF_19)**]** | Basal-type | neg | neg | neg | High | E-cadherin | CD44  EGFR  N- &P-cadherin | HRAS  PIK3CA  P53 |
