# Supplementary figures and images for: T lymphocytes facilitate brain metastasis of breast cancer by inducing Guanylate-Binding Protein 1 expression
Source: Acta Neuropathol. 2018 Jan 19;135(4):581–99. doi: 10.1007/s00401-018-1806-2 (PMC5978929; doi:10.1007/s00401-018-1806-2)

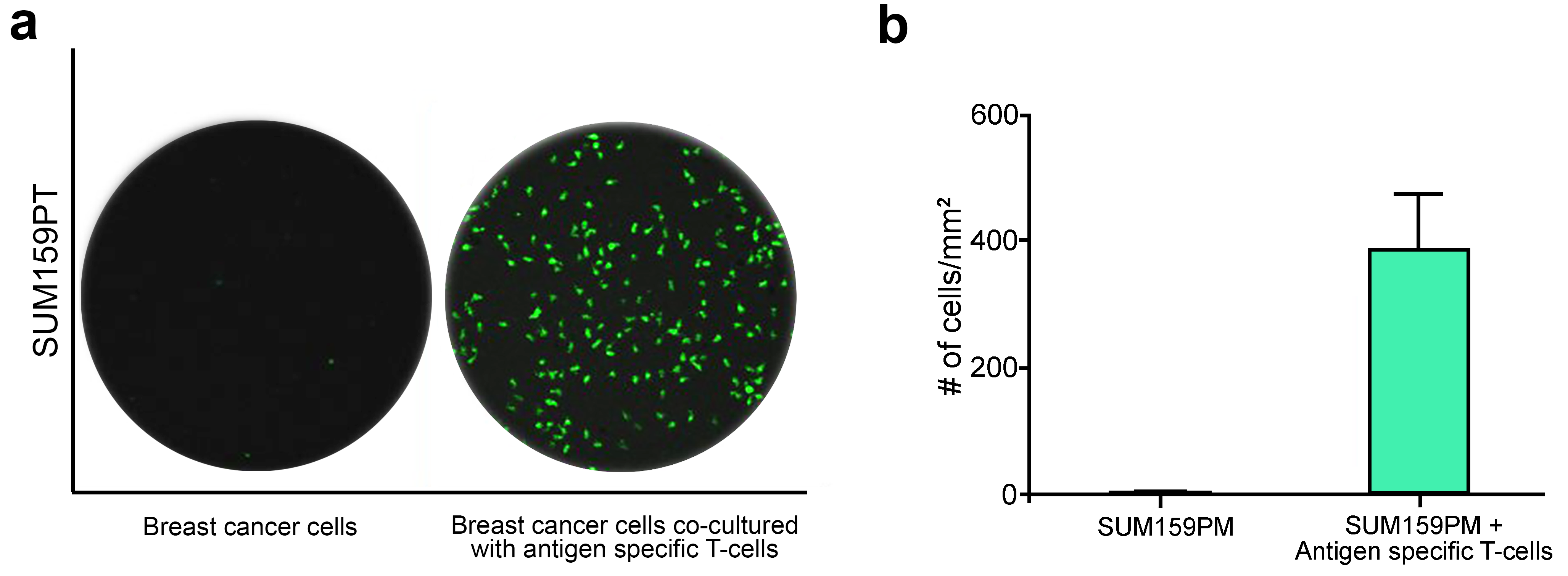

Supplement: Supplementary file 2 — Supplementary material 2 (TIFF 3705 kb). Supplementary Fig. 1: T cells facilitate breast cancer cells to cross the BBB in an antigen-independent fashion. a SUM159PT breast cancer cell line that express the cognate antigen MAGE-C2/HLA-A2 was co-cultured with antigen-specific T cells (CD3+ T lymphocytes transfected with MAGE-C2/HLA-A2 vector). A similar facilitating effect was observed. b Quantitative results of A. Error bars indicate standard deviation [file 401_2018_1806_MOESM2_ESM.tif]
